# *Arabidopsis* HECT and RING-type E3 Ligases Promote MAPKKK18 Degradation to Regulate Abscisic Acid Signaling

**DOI:** 10.1093/pcp/pcad165

**Published:** 2023-12-28

**Authors:** Małgorzata Tajdel-Zielińska, Maciej Janicki, Małgorzata Marczak, Agnieszka Ludwików

**Affiliations:** Laboratory Biotechnology, Institute of Molecular Biology and Biotechnology, Faculty of Biology, Adam Mickiewicz University Poznan, Uniwersytetu Poznańskiego 6, Poznań 61-614, Poland; Laboratory Biotechnology, Institute of Molecular Biology and Biotechnology, Faculty of Biology, Adam Mickiewicz University Poznan, Uniwersytetu Poznańskiego 6, Poznań 61-614, Poland; Laboratory Biotechnology, Institute of Molecular Biology and Biotechnology, Faculty of Biology, Adam Mickiewicz University Poznan, Uniwersytetu Poznańskiego 6, Poznań 61-614, Poland; Laboratory Biotechnology, Institute of Molecular Biology and Biotechnology, Faculty of Biology, Adam Mickiewicz University Poznan, Uniwersytetu Poznańskiego 6, Poznań 61-614, Poland

**Keywords:** ABA, E3 ligases, MAP kinase cascades, MAPKKK17/18, Ubiquitination, UPS

## Abstract

Mitogen-activated protein kinase (MAPK) cascades are conserved signaling pathways that transduce extracellular signals into diverse cellular responses. *Arabidopsis* MAPKKK18 is a component of the MAPKKK17/18-MKK3-MPK1/2/7/14 cascades, which play critical roles in abscisic acid (ABA) signaling, drought tolerance and senescence. A very important aspect of MAP kinase signaling is both its activation and its termination, which must be tightly controlled to achieve appropriate biological responses. Recently, the ubiquitin-proteasome system (UPS) has received increasing attention as a key mechanism for maintaining the homeostasis of MAPK cascade components and other ABA signaling effectors. Previous studies have shown that the stability of MAPKKK18 is regulated by the UPS via the ABA core pathway. Here, using multiple proteomic approaches, we found that MAPKKK17/18 turnover is tightly controlled by three E3 ligases, UPL1, UPL4 and KEG. We also identified lysines 154 and 237 as critical for MAPKKK18 stability. Taken together, this study sheds new light on the mechanism that controls MAPKKK17/18 activity and function.

## Introduction

The mitogen-activated protein (MAP) kinase (MAPK) cascade typically consists of three types of kinases, MAPKKK, MAPKK and MAPK, which are activated sequentially by phosphorylation. MAP cascades play a crucial role in the transduction of extracellular signals into various intracellular responses to regulate cell functions (Ichimura et al. 2002). In plants, the MAPK pathway plays a crucial role in effective tolerance, adaptation and survival of adverse conditions, including biotic and abiotic stresses. Due to its involvement in so many different processes, the MAPK signaling pathway cross-talks with several other signaling pathways, including hormonal signaling. Among these, abscisic acid (ABA) signaling is of particular importance, as it is known to coordinate responses and adaptation to environmental stresses.

Among the three major MAPK families, MAPKKK is the largest (80 members in *Arabidopsis*) and the most heterogeneous group. To date, only a few have been described and implicated in ABA signaling (Danquah et al. 2015, Jagodzik et al. 2018). One of the best characterized MAPKKKs mediating ABA is MAPKKK18. We previously showed that an ABA-activated MAPKKK18 is involved in several physiological processes regulated by ABA, including seed germination, stomatal development and function (Danquah et al. 2015; Mitula et al. 2015). In addition, a complete MAPK module starting with MAPKKK18 has been identified, the MAP3K17/18-MKK3-MPK1/2/7/14 cascade, which modulates two other processes, leaf senescence (Matsuoka et al. 2015) and response to drought stress (Li et al. 2017).

Very important aspects of MAPK signaling are both its activation and its termination, which must be tightly controlled to achieve an appropriate integrated biological response to different stimuli (Witzel et al. 2012). The main mechanism that negatively regulates signaling through the MAPK cascade is the dephosphorylation of its components by protein phosphatases (Fuchs et al. 2013). In recent years, however, the ubiquitin-proteasome system (UPS) has attracted considerable research attention as another key mechanism involved in maintaining the homeostasis of MAPK cascade components and other ABA signaling effectors.

Ubiquitination, one of the most common post-translational modifications, not only acts as a signal for proteasomal degradation, but also affects all aspects of cellular signaling and protein homeostasis (Glickman and Ciechanover 2002, Pickart and Fushman 2004). Functional consequences of ubiquitination of ABA effectors include changes in localization, activity, and interaction network (Raed Al-Saharin et al. 2022, Wang et al. 2023). The process of ubiquitination involves a three-step enzymatic cascade: activation of ubiquitin by an E1-activating enzyme, transfer of activated ubiquitin to an E2-conjugating enzyme, and finally attachment of ubiquitin to a specific lysine residue of the target protein. This final step is catalyzed by an E3 ubiquitin ligase, which is responsible for the specificity of the recognized substrate protein and mediates protein ubiquitination (Vierstra 2009, Lee and Kim 2011, Yu et al. 2021). In *Arabidopsis*, more than 1,400 E3 ligases have been identified and classified into four major families. Based on the mechanism of ubiquitin transfer from the E2 enzyme to the target protein, E3 ligases have been divided into the following four groups RING (Really interesting new gene), HECT (homologous to E6-AP C-terminus), U-box and finally CRL (Cullin-RING Ligases) (Al-Saharin et al. 2022). Among them, the RING family has been intensively studied in different stages of ABA signaling (Yu et al. 2021), compared to the least functionally characterized HECT group.

In our previous work, we found that the PP2C phosphatase ABI1 affects MAPKKK18 function by inhibiting its kinase activity and targeting MAPKKK18 for degradation via the UPS (Mitula et al. 2015, Tajdel et al. 2016). However, the exact mechanism of MAPKKK18 protein turnover remains to be elucidated. Using different proteomic approaches, we found that the protein level of MAPKKK18 (as well as MAPKKK17) is tightly controlled by three E3 ligases: UPL1, UPL4 and KEG, belonging to the HECT and RING families respectively. We also identified lysine residues 154 and 237 (K154 and K237) as critical ubiquitin acceptor sites for MAPKKK18. Taken together, our results provide important insights into the regulatory mechanisms involved in the control of MAPKKK17/18 activity and function.

## Results

### Identification of E3 ligases in MAPKKK18 complexes

Our previous study showed that MAPKKK18 is regulated by the ubiquitin proteasome pathway; however, the underlying mechanism involved in regulating MAPKKK18 turnover is unknown (Mitula et al. 2015). To gain insight into the regulation of MAPKKK18 by the UPS, we searched for proteins interacting with MAPKKK18 from the proteasome pathway using the GST pull-down approach coupled with LC-MS/MS. The recombinant GST-MAPKKK18 was incubated with the total-cell extract isolated from one-week-old *Arabidopsis* wild-type (WT) Col-0 plants. GST-CDPK34 was used as a control for nonspecific-binding kinase. The proteins bound by GST–MAPKKK18 were identified by LC-MS/MS. The list of all proteins identified in GST–MAPKKK18 complexes, and the controls can be found in the **Supplementary Table 1**. Importantly, this method allowed the identification of proteins potentially involved in the regulation of MAPKKK18 abundance, i.e. UPS-related proteins. These include components of the 26S proteasome, including proteins that make up the 19S regulatory unit (RPN5a) and the base of the 19S regulatory unit (RPT5a), as well as protein that are part of the alpha ring of the 20S proteasome proteolytic unit PAF2 (**Supplementary Table 1**). However, the most important proteins identified in MAPKKK18 complexes are E3 ligases, a RING-type KEG ubiquitin ligase and two HECT-type E3 ubiquitin ligases: UPL1 and UPL4 (**Supplementary Table 1**).

### KEG interacts with MAPKKK18 and promotes its turnover

**Fig. 1:**
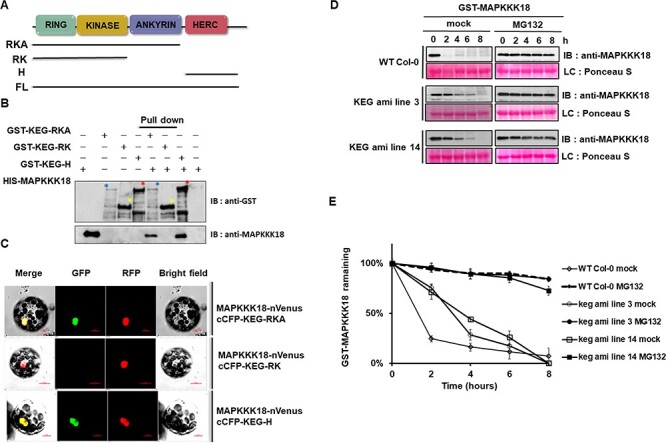
KEG E3 ligase interacts with MAPKKK18 both *in vitro* and *in vivo* and contributes to the turnover of MAPKKK18.

KEG appears to play multiple roles in plant development and is involved in ABA signaling. The E3 ligase KEG ubiquitinates effectors of the ABA signaling pathway and promotes their degradation in vivo (Stone et al. 2006, Chen et al. 2013a, Liu and Stone 2014). The presence of KEG in GST–MAPKKK18 complexes and the association of KEG with ABA signaling prompted us to investigate whether KEG regulates the degradation of MAPKKK18. To determine the underlying mechanism by which MAPKKK18 stability is regulated by KEG ligase, we investigated a possible interaction between MAPKKK18 and KEG. Since KEG is an unusually large protein (1,625 amino acids), we first performed a pull-down assay using KEG deletion fragments to test whether KEG associates with MAPKKK18. The KEG protein consists of the following RING domain (R), which is involved in ubiquitin transfer (Stone et al. 2006), kinase domain (K), and finally two other domains, a series of ankyrin repeats (A) and 12 HERC2-like repeats (H), both of which can act as substrate binding modules (Stone et al. 2006). Three truncated versions of GST-KEG were used in the pull-down experiment together with 6 × His-MAPKKK18 (**Fig. 1A**). These include KEG fragment containing RING and kinase domain (GST-KEG-RK); RING, kinase domain and ankyrin repeats (GST-KEG-RKA) and finally N-terminal HERC2-like repeats only (GST-KEG-H). The scheme and description of the truncated versions of the KEG protein are shown in **Fig. 1A**. GST-KEG-RKA fragments containing the ankyrin repeats and GST-KEG-H were able to pull-down 6×HIS-MAPKKK18 (**Fig. 1B**), whereas GST-RK was not able to pull-down 6×HIS-MAPKKK18 (**Fig. 1B**).

(A) Schematic representation of the KEG domains and truncated versions of the protein used in the pull-down assay and BiFC analysis. The full-length (FL) KEG E3 ligase consists of four domains: the N-terminal RING domain of KEG (R), the kinase domain (K), the ankyrin repeats (A) and the C-terminal HERC2-like repeats (H). The following three KEG deletion fragments were used: (1) RING domain, kinase domain and ankyrin repeats (RKA); (2) RING domain and kinase domain (RK) and (3) HERC2-like repeats (H); (B) Detection of direct interaction between MAPKKK18 and truncated versions of KEG by GST pull-down assay. After pull-down with GST-KEG fragments, proteins were resolved on 10% SDS-PAGE, transferred to PDVF membrane and immunoblotted. Anti-GST was used to detect ‘bait’ proteins immobilized on glutathione agarose beads—KEG deletion fragments (GST-KEG-RKA—blue asterisk, GST-KEG-RK—yellow asterisk and GST-KEG-H—red asterisk; top panel). Anti-MAPKKK18 was used to detect the eluted ‘prey’ protein 6xHIS-MAPKKK18 kinase (bottom panel). Lanes 1, 2, 3 and 4 show the input. Lanes 5 and 7 show the pull-down of MAPKKK18 through the GST-KEG-RKA and GST-KEG-H domains. Lane 7 is the resin control where HIS-MAPKKK18 was incubated with the glutathione-agarose beads. In the resin control, the same aliquot of HIS-MAPKKK18 and the same washing procedures were used as in the pull-down reaction. The presence or absence of a particular protein in each reaction is indicated by ‘+’ and ‘-’; (C) Confirmation of the interaction between MAPKKK18 and KEG deletion fragments by multicolor bimolecular fluorescence complementation (mcBiFC) assay in *Arabidopsis* mesophyll protoplasts. The plasmid pairs MAPKKK18-nVenus and cCFP-KEG-RKA, MAPKKK18-nVenus and cCFP-KEG-H, MAPKKK18-nVenus and cCFP-KEG-RK were transiently co-expressed in mesophyll protoplasts of *Arabidopsis* leaves. Co-expression of the fusion proteins results in a BiFC signal in the nucleus (MAPKKK18-nVenus with cCFP-KEG-RKA and MAPKKK18-nVenus with cCFP-KEG-H) and in the cytoplasm (MAPKKK18-nVenus with cCFP-KEG-RKA). Co-transformations of the empty vector pS6C1C or pS5C4N with the fusion proteins MAPKKK18-nVenus or cCFP-KEG-RKA/RK/H were used as controls. Expression of RFP-C-labeled CBP20 was used as a marker for nuclear localization; scale bars: 10 μm; (D) Cell-free degradation assay for GST–MAPKKK18. Recombinant GST–MAPKKK18 was incubated for 0–8 h with total proteins extracted from 1 week-old *Arabidopsis* seedlings of lines KEG-ami3/14 and wild-type (Col-0 WT); treated (lanes labeled as +MG132) or untreated with 50 μM MG132 (lanes labeled as mock). GST–MAPKKK18 protein levels at the indicated time points (0, 2, 4, 6 and 8 h) were examined by Western blotting with anti-MAPKKK18 antibody (top panel). Immunoblots are representative of at least three replicates. Ponceau S membrane staining of the large subunit of Rubisco (band at 55 kDa) indicates equal loading of proteins (lower panel); (E) The band density corresponding to GST–MAPKKK18 (60 kDa) was determined using ImageJ. The bars indicate the standard deviation of at least three replicates.

To confirm the interaction between MAPKKK18 and KEG segments in planta, we used a multicolor bimolecular fluorescence complementation (mcBiFC) assay in *Arabidopsis* protoplasts (**Fig. 1C**). In this mcBiFC approach, *A. thaliana* protoplasts were co-transformed with MAPKKK18 fused to the N-terminal part of the fluorescent protein Venus (nVenus) and KEG constructs fused to the C-terminal fragment of the CFP protein (cCFP). The CBP20-RFP construct was used as a transformation control and as a nuclear marker. A BiFC signal was detected in protoplasts co-expressing MAPKKK18-nVenus and two previously described KEG fragments, cCFP-KEG-RKA and cCFFP-KEG-H, confirming that MAPKKK18 interacts with these two fragments (**Fig. 1C**). As a negative control, four co-transformations of protoplasts were prepared, including co-transformations with individual KEG deletion fragments fused to cCFP (cCFP-KEG-RKA, cCFP-KEG-RK and cCFP-KEG-H) and the empty vector pS5N4N encoding nVenus; and co-transformations of the empty vector pS6C1C-encoding cCFP with MAPKKK18-nVenus (**Supplementary Fig. 1**).

The interaction between KEG E3 ubiquitin ligase and MAPKKK18 (**Fig. 1B, C**) suggests that KEG is involved in MAPKKK18 degradation. To test this hypothesis, we performed an in vitro cell-free degradation assay. The recombinant GST–MAPKKK18 protein was mixed with protein extracts from the KEG knockout lines (KEG amiRNA line 3 and 14; Pauwels et al. 2015) and WT Col-0 plants, with and without MG132, respectively. As shown in **Fig. 1D, E**, the degradation of GST-MAPKKK18 protein was significantly delayed in the KEG amiRNA lines 3 and 14 without MG132 compared to the WT Col-0 extract without MG132 treatment. The half-life of GST–MAPKKK18 incubated with WT Col extract without MG132 treatment was less than 2 h (*t*1/2 < 2 h). The half-life of GST-MAPKKK18 increased to ∼ 4 h in plant extracts of KEG amiRNA line 14 (*t*1/2 = 4 h) and to ∼ 3 h in plant extracts of KEG amiRNA line 3 (*t*1/2 = 3 h) (**Fig. 1D, E**). These results suggest that KEG E3 ligase regulates the stability of MAPKKK18.

### Two HECT-type ligases, UPL1 and UPL4 are involved in MAPKKK18 degradation

**Fig. 2:**
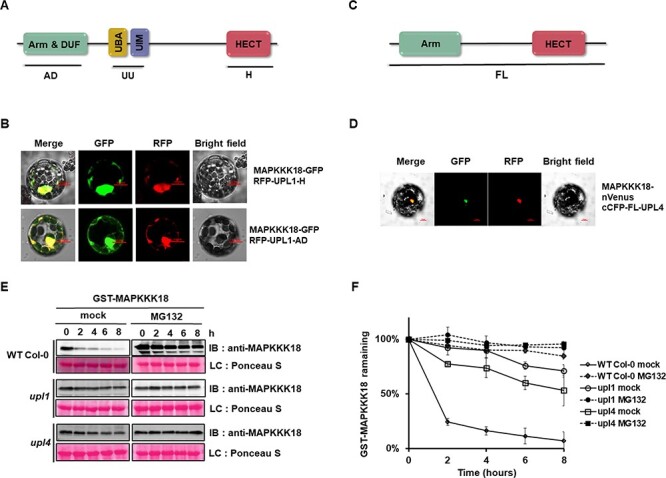
HECT-type E3 ubiquitin ligases UPL1 and UPL4 are involved in the proteasomal degradation of MAPKKK18.

The presence of two HECT-type E3 ubiquitin ligases, UPL1 and UPL4, in GST–MAPKKK18 complexes suggested their involvement in the ubiquitination of MAPKKK18. Again, it was technically difficult to construct an expression vector to overexpress UPL1 due to its large size (3,681 amino acids). Therefore, to verify whether UPL1 interacts with MAPKKK18 in planta, for Förster resonance energy transfer by fluorescence lifetime imaging (FRET-FLIM) analyses, we used fragments of UPL1 proteins consisting only of the HERC2-like repeat domain (H), Armadillo (ARM)-type fold & domain of unknown function (DUF) domain (AD) and ubiquitin-associated & ubiquitin Interacting Motif (UBA&UIM) domains (UU) (**Fig. 2A**). The analyzed pairs of proteins were colocalized; however, the FRET was negative, in this configuration (**Fig. 2B**).

(A) Domain architectures of full-length (FL) UPL1 and UPL1 truncated fragments used for FRET-FLIM analysis. The following domains are included in the UPL1 protein structure: the armadillo-type fold (A); the DUF domain (D); the ubiquitin associated domain (UBA); the ubiquitin interacting motif (UIM); and the homologous to the E6-AP carboxyl terminus domain (HECT). Four UPL1 deletion fragments were used in the analyses: (1) armadillo-type fold and DUF domain (AD); (2) ubiquitin-associated domain and ubiquitin-interacting motif (UU); (3) HECT domain (H); (4) ubiquitin-associated domain (inclusive) to HECT domain (UH); (B) Co-localization of MAPKKK18 with truncated versions of UPL1 in the *Arabidopsis* protoplast. Plasmid pairs MAPKKK18–GFP and RFP-UPL1-AD, MAPKKK18–GFP and RFP-UPL1-UU, MAPKKK18–GFP and RFP-UPL1-H, MAPKKK18-nVenus and cCFP-KEG-Herc, MAPKKK18-nVenus and cCFP-KEG-RK were transiently co-expressed in *Arabidopsis* mesophyll protoplasts. Fluorescence signals of MAPKKK18 fused to GFP are shown in green. Fluorescence signals of UPL1 deletion fragments fused to RFP are shown in red. Colocalization of MAPKKK18 with specific subunits of UPL1 occurs in the nucleus and cytoplasm and is shown in yellow (merge panel); scale bars: 10 μm; (C) Domain architectures of the full-length (FL) UPL4 protein. Two domains are included in the UPL4 protein structure: the N-terminal armadillo-type fold (A); the C-terminal HECT domain (HECT). Full-length UPL4 was used for BiFC; (D) Identification of MAPKKK18-UPL4 in vivo interaction using the mcBiFC assay in *Arabidopsis* mesophyll protoplasts. Construct pairs of MAPKKK18-nVenus and cCFP-UPL4, empty nVenus vector and cCFP-UPL4, nVenus-MAPKKK18 and empty cCFP vector, empty nVenus vector and empty cCFP vector were co-expressed in protoplasts. Green BiFC fluorescence was observed in the nucleus and is indicated by the fluorescence signal. Scale bars: 10 μm; (E) Cell-free degradation assay of GST–MAPKKK18 in *upl1* and *upl4* plant. Recombinant GST–MAPKKK18 was incubated with protein extracts isolated from 1-week-old *Arabidopsis* seedlings of lines upl1 and wild-type (Col-0 WT); treated (lanes labeled as +MG132) or untreated with 50 μM MG132 (lanes labeled as mock). To monitor the level of GST–MAPKKK18 at the indicated time points (0, 2, 4, 6 and 8 h), immunoblotting with anti-MAPKKK18 was used (top panel). Immunoblots are representative of at least three replicates. Ponceau S membrane staining of the large subunit of Rubisco (band at 55 kDa) indicates equal loading of proteins (lower panel); (F) The band density corresponding to GST–MAPKKK18 (60 kDa) was determined using ImageJ. The bars indicate the standard deviation for at least three replicates.

To verify the interaction between MAPKKK18 and UPL4 in planta, we again performed a multicolor bimolecular fluorescence complementation (mcBiFC) assay in *Arabidopsis* protoplast cells (**Fig. 2C, D**) using CBP20-RFP as a transformation control and simultaneously as a nuclear marker. When MAPKKK18 fused to the N-terminal part of the fluorescent protein Venus (nVenus) and UPL4 fused to the C-terminal part of the CFP protein (cCFP) were transiently co-expressed in the protoplast, we observed a BiFC signal detected in the nucleus (**Fig. 2D**). An interaction between MAPKKK18 and UPL4 ligase suggests that this E3 ligase may regulate the stability of MAPKKK18.

To test whether UPL1 or UPL4 affects MAPKKK18 degradation, we again used cell-free degradation assays. In these assays, recombinant GST–MAPKKK18 was incubated in the presence of ATP with total protein extracts from WT Col-0 or *upl1, upl4*. The degradation rate of GST–MAPKKK18 was much slower in extracts from the two knockout lines, *upl1* and *upl4*, than from WT Col-0. The half-life of GST–MAPKKK18 incubated in the *upl1* extract is more than 8 h (*t*1/2 > 8 h), whereas the half-life of GST–MAPKKK18 incubated in the *upl4* extract is ∼ 8 h (*t*1/2 ∼ 8 h) (**Fig. 2E, F**). These results support the notion that these two HECT-type ligases, UPL1/4, mediate MAPKKK18 degradation in a 26S proteasome-dependent manner.

### Lysines 154 and 237 are involved in MAPKKK18 degradation

The next step was to determine which lysine(s) in the MAPKKK18 sequence might be required for MAPKKK18 ubiquitination. There are 15 lysine residues in MAPKKK18. To narrow down the possible ubiquitination sites of MAPKKK18, we used several ubiquitination site prediction tools available online, including UbPred (Radivojac et al. 2010), UbiProber (Chen et al. 2013a), hCKSAAP_UbSite (Chen et al. 2013a) and BDM-PUB (Li et al. 2009). Six lysine residues (7, 32, 146, 154, 245 and 246) with high confidence scores were identified (**Supplementary Table 2**).

**Fig. 3:**
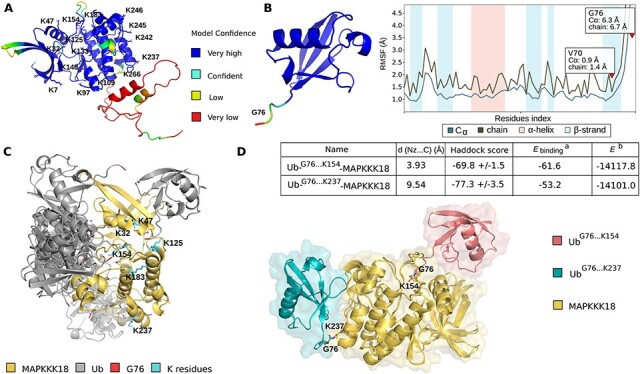
In silico structural prediction of potential ubiquitinated lysine residues in MAPKKK18.

To identify the key lysine residues that can be ubiquitinated, we also analyzed the structural capabilities of potential MAPKKK18 ubiquitination sites using a molecular modeling approach. Ubiquitination is a post-translational modification in which the ubiquitin molecule forms a covalent bond between the carboxyl group of the glycine residue (G76) of Ub and the lysine amino group of the target protein. The model of the MAPKKK18 protein structure was developed using AlphaFold2 (Jumper et al. 2021). The resulting structure was minimized in the OPLS4 force field (Lu et al. 2021). The C-terminal region of MAPKKK18 was neglected in all simulations according to the very low score of modeling predicted by Artificial Intelligence (**Fig. 3A**). The same approach was performed with the Ub protein structure, but the C-terminal region was included (**Fig. 3B**). To determine which lysine residues of MAPKKK18 can be modified by ubiquitin attachment, the MAPKKK18–Ub complex was designed. A multi-step approach was used to simulate the formation of the covalent bond between Ub and MAPKKK18. First, blind docking was performed using the Cluspro webserver (Kozakov et al. 2017).

(A–B) Structures of MAPKKK18 (A) and of the Ub protein (B) predicted using AlphaFold2. The AlphaFold pre-residue model confidence score (pLDDT) has been used to highlight the scored structures; plot showing the positional fluctuations of all amino acids for the Ub protein based on the trajectory from MD simulations; RMSF is the root mean square fluctuation calculated for the side chain of a given residue (chain) or from the trace Cα. The plot shows a highly flexible region on the C-terminal side of Ub. (C) Initial docking results from Cluspro webserver with preselected K residues marked as potential ubiquitination sites. (D) Best models of Ub-MAPKKK18 obtained from structural prediction with K157 and K237 highlighted as potential Ub targets. The table summarises the best selected models of MAPKKK18-UBQ complexes, including the distance in Å between the nitrogen atom (Nz) of the MAPKK18 lysine residue and the alpha carbon atom (C) of the ubiquitin G76 residue, the Haddock score, (a) MM-GBSA-binding energy (kcal/mol) estimation evaluated from Prime module. (b) Energy (kcal/mol) of minimized Ub-MAPKKK18 structure obtained from Prime module.

MAPKKK18–Ub complexes were evaluated and ranked according to a balanced scheme (Kozakov et al. 2017). Based on the results obtained, lysine(s) where the distance from G76 was smaller than 10 Å were considered as potentially ubiquitinated. From the first docking step, K residues (32, 47, 125, 154, 183 and 237) were selected as potential binding sites of the MAPKKK18 target protein (**Fig. 3C**). The binding site of Ub protein was selected as residue G76. The selected binding sites were used as constraints to construct the models of MAPKKK18–Ub complexes using Haddock (Van Zundert et al. 2016, Honorato et al. 2021). For this step, the structure of MAPKKK18 and 14 conformers of Ub were used. The Ub conformers were obtained from the trajectory after MD simulation. The inclusion of additional conformers makes our docking protocol more flexible, especially considering the C-terminus of Ub, which is structurally disordered (**Fig. 3B**) and directly involved in binding formation. The obtained macromolecular docking results were clustered and ranked based on the Haddock scoring function, and from each set the best model of the Ub–MAPKKK18 structural complex was selected. The distance between G76 and each K (possibility of covalent bond formation in each evaluated Ub–MAPKKK18 complex) was measured and after this analysis structures that could not form a covalent bond due to steric clashes or that involved the lysine residue in another interaction were rejected.

For the best models, the binding energy, and the energy of Ub–MAPKKK18 were calculated after manual formation of the covalent bond between G76 and the K residues. From the pre-selected K32, K47, K125, K154, K184 and K237 according to the structural bioinformatics analysis mentioned above, lysine 154 and 237 were selected as the most potential targets for ubiquitination (**Fig. 3D**). Altogether, based on the in silico approach, K residues (7, 32, 146, 154, 237, 245 and 246) were selected for experimental verification as potential ubiquitination sites. K154 was identified by both predictions, K (7, 32, 146, 245 and 246) was selected according to ubiquitination site prediction tools and K237 was obtained by structural analysis only.

**Fig. 4:**
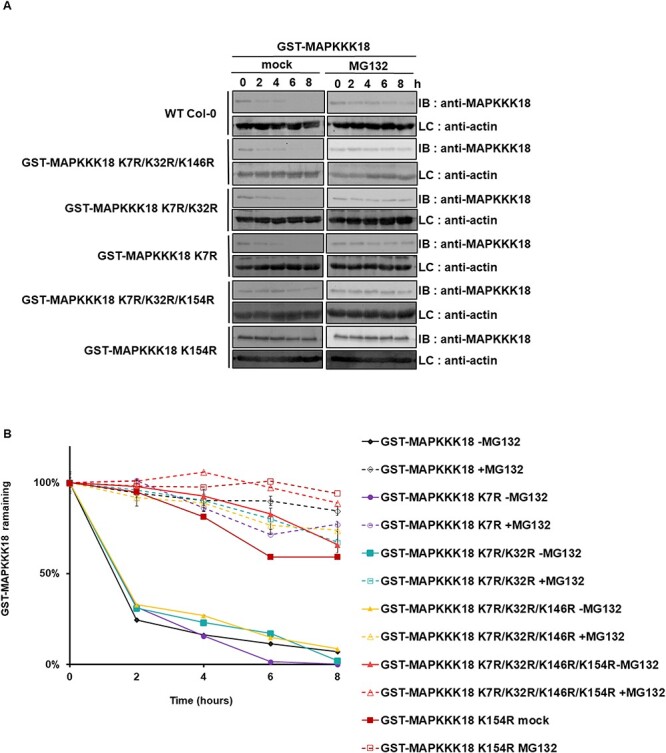
Lysine residue 154 in the MAPKKK18 sequence identified as potential ubiquitination sites.

**Fig. 5:**
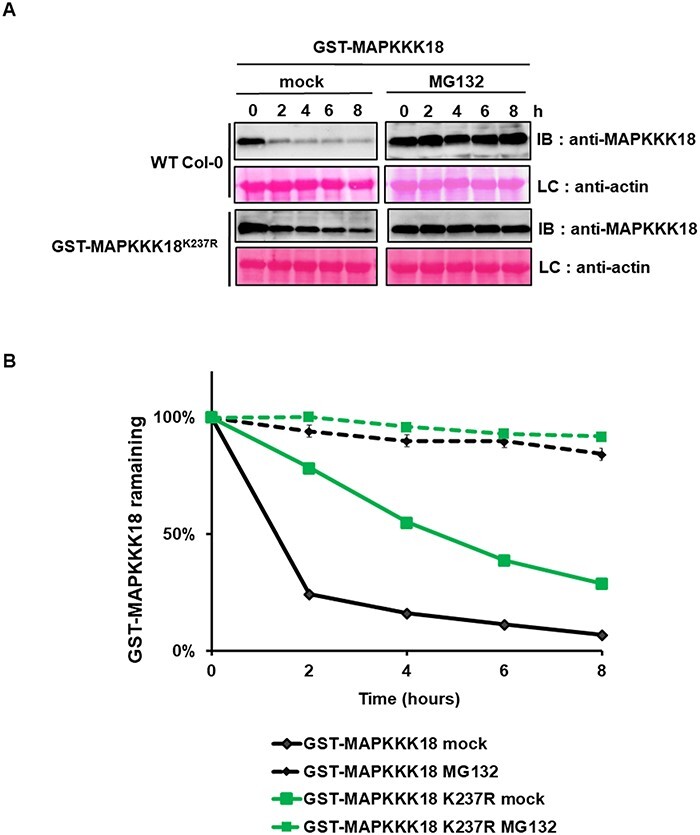
Lysine residue 237 in the MAPKKK18 sequence identified as potential ubiquitination sites.

For the experimental determination of ubiquitination sites, lysine residues were mutated to arginine (K to R mutant versions of the proteins) to prevent ubiquitination at the corresponding site (Gilkerson et al. 2015). Seven candidate lysine residues of MAPKKK18 were analyzed (K7R, K32R, K146R, K154R, K237 R, K245R and K246R) and cell-free degradation assay was performed to monitor the protein stability of MAPKKK18. We incubated recombinant non-mutated GST–MAPKKK18 and lysine-to-arginine mutants of GST–MAPKKK18 protein with total plant extract WT Col-0 in the presence of ATP, with and without MG132. The half-life of GST–MAPKKK18 containing K7, K32, K146R, K245R and K246 mutations was like that of wild-type MAPKKK18 in plant extracts not treated with MG132 and was less than 2 h (*t*1/2 < 2 h) (**Fig. 4A, B**; **Fig. 5A, B**), **Supplementary Fig. 2**). The degradation rate of GST–MAPKKK18 containing mutations of K154 (GST-MAPKKK18 K7R-K32R-K154R and GST-MAPKKK18 K154R) and K237, respectively, was significantly delayed in untreated plant extracts compared to wild-type MAPKKK18 in untreated plant extracts. The half-life of GST–MAPKKK18 containing the K154 mutation was more than 8 h (*t*1/2 > 8 h) (**Fig. 4 A, B**), while the *t*1/2 of MAPKKK18 containing K237 R was significantly longer than 4 h (*t*1/2 > 4 h) (**Fig. 5A, B**). These results indicate that lysine 154 and 237 are involved in the proteasomal degradation of MAPKKK18.

(A) Cell-free degradation assay for GST–MAPKKK18 single and multiple site-specific mutants K7R, K7R/K32R, K7R/K32R/K146R, K7R/K32R/K146R/K154R, K154R, K245R, K246R in WT plants. The recombinant GST–MAPKKK18 and its mutant versions were incubated with total plant extract isolated from 7-day-old seedlings in the absence (mock) or presence (+) of MG132. The level of each recombinant protein was monitored by immunoblotting with anti-MAPKKK18 at the indicated time points. (B) The amount of GST–MAPKKK18 and its mutant versions was determined using ImageJ and plotted. Error bars indicate standard deviation.

(A) Cell-free degradation assay of site-specific GST–MAPKKK18 mutant K237 R in WT plants. GST–MAPKKK18K237 R and GST–MAPKKK18 proteins were added to total plant extract prepared from 7-day-old seedlings. The levels of each recombinant protein were checked by Western blotting using specific anti-MAPKKK18 antibodies at the indicated time points. The reaction was performed in the absence (mock) or presence (+) of MG132. (B) The amount of GST–MAPKKK18 and its mutant version was determined by ImageJ using the signal intensity at the indicated time and plotted. Error bars indicate standard deviation.

### Prediction of the UBC10–Ub–KEG–MAPKKK18 complex

E1, E2 and E3 ligases are involved in the process of target protein ubiquitination (Liu et al., 20–21). Our study confirmed that KEG is an E3 ligase for MAPKKK18 (**Fig. 1**). As KEG is an unusually large protein, KEG deletion fragments were used to validate the interaction between MAPKKK18 and the ANK repeats and the HERC domain. KEG contains a RING-type domain, so the mechanism of Ub transfer to the target protein involves E2 with conjugated ubiquitin and E3 binding a substrate protein. The transfer of Ub occurs directly from the E2 ligase to the target protein. Based on these data, UBC10–Ub–KEG–MAPKKK18 was constructed using a multi-step molecular modeling approach. In the first step, the complex model of UBC10–Ub–KEG was predicted using the docking protocol described in **Supplementary Fig. 3**. For the best scoring, UBC10–Ub–KEG complex model (**Supplementary Fig. 4A**), docking of MAPKKK18 was performed. The simulation was performed using two approaches based on the identification of K154 and K237 involved in MAPKKK18 degradation. Selected K residues and C85 of KEG–UBC10–Ub were selected as active residues during docking. All models obtained were ranked according to the Haddock score and followed by visual inspection to check the possibility of forming a covalent bond between the selected lysine residue (K154, K237) and G76 (distance between G76 and K residue). After this analysis, models that could not form a covalent bond were discarded. The final step was to select models based on experimental data, which in this study showed that MAPKKK18 can interact with ANK repeats and the HERC domain. Based on this, we show the best scoring models UBC10–Ub–KEG–MAPKKK18K154 (**Supplementary Fig. 4B**) and UBC10–Ub–KEG–MAPKKK18K237 (**Supplementary Fig. 4C**). The results show that the obtained structures of UBC10–Ub–KEG–MAPKKK18, with the high score reproducing the experimental data, where MAPKKK18 was shown to interact with ANK repeats and the HERC domain (**Supplementary Fig. 4B, C**).

### UPL1, UPL4 and KEG regulate MAPKKK17 stability

**Fig. 6:**
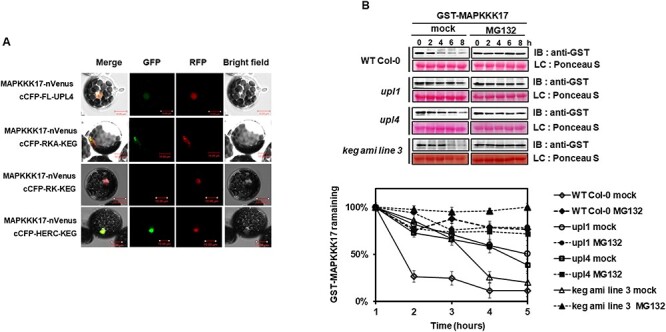
MAPKKK17 stability is regulated by proteasomal degradation mediated by UPL1, UPL4 and KEG E3 ligases.

MAPKKK17 and MAPKKK18 are closely related kinases with redundant functions in ABA signaling and response (Zhang and Zhang 2022). To gain insight into the regulation of MAPKKK17, we examined its possible regulation by UPL1, UPL4 and KEG. As shown in **Fig. 6**, MAPKKK17 also interacts with UPL4 and KEG-H fragment (**Fig. 6A**). In the cell-free assay, we observed a significant delay in MAPKKK17 degradation in the *upl1, upl4* and KEG amiRNA lines 3 and 14 without MG132 compared to WT Col-0 (**Fig. 6B**), suggesting that all three E3 ligases also regulate MAPKKK17 stability.

(A) BiFC assay in *Arabidopsis* mesophyll protoplasts showing the identification of the *in vivo* interaction between MAPKKK17 and UPL4, and also between MAPKKK17 and KEG fragments. The four plasmid pairs: MAPKKK17-nVenus and cCFP-UPL4; MAPKKK17-nVenus and cCFP-KEG-RKA; MAPKKK17-nVenus and cCFP-KEG-H; MAPKKK17-nVenus and cCFP-KEG-RK were transiently co-expressed in mesophyll protoplasts of *Arabidopsis* leaves. Green BiFC fluorescence was observed in the nucleus. RFP-C-labeled CBP20 was used as a marker for nuclear localization. Scale bars: 10 μm. (B). Cell-free degradation assay for GST-MAPKKK17. Recombinant GST–MAPKKK17 was incubated with total protein extracts isolated from 7-day-old *Arabidopsis* seedlings of lines *upl1, upl4*, KEG-ami3 and WT Col-0; treated or untreated with 50 μM MG132. GST–MAPKKK17 protein levels at the indicated time points (0, 2, 4, 6 and 8 h) were detected by Western blotting with anti-GST antibody (top panel). Immunoblots are representative of at least three replicates. To show equal loading, membranes were stained with Ponceaus S (lower panel). The density of the GST–MAPKKK17 band (65 kDa) was determined using ImageJ. Bars represent the standard deviation of at least three replicates.

### UPL1, UPL4 and KEG regulate ABA-dependent germination

**Fig. 7:**
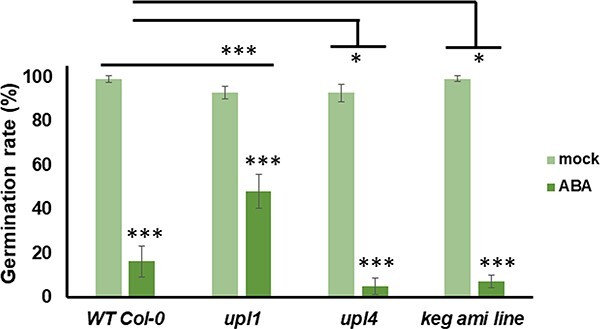
UPL1 affect early developmental processes regulated by ABA.

Previous data using KEG T-DNA insertion lines indicate that KEG is required for ABA signaling and postgerminative growth of *Arabidopsis* seedlings (Stone et al. 2006). The biological functions of UPL1 and UPL4 remain largely unknown. To investigate the physiological role of the investigated E3 ligases, we determined whether UPL1 and UPL4 are involved in plant ABA responses. In the germination test carried out under controlled conditions, the germination percentages of the genotypes studied were similar. However, at high concentrations of 0.5 µM (+)ABA, *upl1* shows robust insensitivity to 0.5 µM (+)ABA, whereas *upl4* and the *keg ami* line show delayed germination to high concentrations of 0.5 µM (+)ABA (**Fig. 7**).

Germination rates of WT Col-0 *upl1, upl4* and *keg-ami3* on 0.5 µM (+) ABA. Seed germination was scored after 7 days. Values are mean ± SE for at least three independent experiments (*n* > 35). *P < 0.01; **P < 0.001; ***P < 0.0001 compared to the control line WT Col-0.

### Discussion

**Fig. 8:**
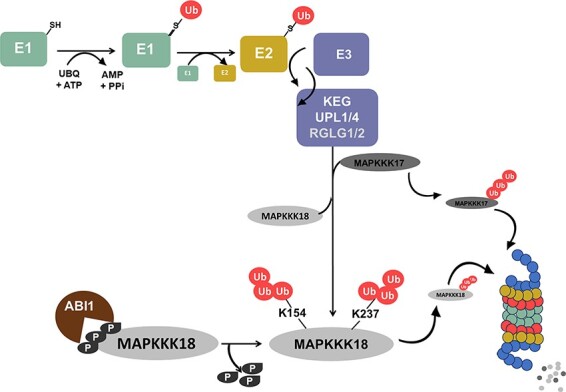
UPS-mediated degradation of MAPKKK17/18.

*Arabidopsis* MAPKKK18, together with MAPKKK17, initiates the MKK3-MPK1/2/7/14 module that mediates ABA response, leaf senescence and drought stress response (Danquah et al. 2015, Matsuoka et al. 2015, Mitula et al. 2015, Li et al. 2017). Previously, we provided evidence that the mechanism of MAPKKK18 signaling inactivation is based on the UPS regulated by ABA via the ABA core pathway (Mitula et al. 2015). The aim of this study was to characterize the regulatory mechanism of MAPKKK18 turnover. As MAPKKK17 is a highly homologous kinase, we included it in the analysis. Here, we present the results indicating that the turnover of MAPKKK17/18 is controlled by three specific E3 ubiquitin ligases, two HECT-type ligases UPL1 and UPL4, and the RING family of KEG ligases (**Figs. 1**, **2** and 6). We also found that lysines 154 and 237 are critical ubiquitin acceptor sites for MAPKKK18 (**Figs. 4**, **5**). Overall, this study provides a mechanistic understanding of how MAPKKK17/18 abundance is regulated by the ubiquitin-proteasome pathway (**Fig. 8**).

MAPKKK18 is targeted for proteasomal degradation by a three-step enzymatic cascade involving E1 ubiquitin-activating enzyme, E2 ubiquitin-conjugating enzyme and finally E3 ubiquitin ligase. As a key enzyme in this process, the E3 ligase is responsible for the specificity of the recognized substrate and the attachment of ubiquitin to a specific lysine residue. We have identified three E3 ligases that can ubiquitinate MAPKKK18, KEG, UPL1 and UPL4. Other known ligases involved in MAPKKK18 turnover are RGLG1/2. Lysine residues 154 and 237 (K154 and K237) have been identified as critical ubiquitin acceptor sites for MAPKKK18. The 26S proteasome recognizes the ubiquitination signal (polyubiquitination), removes it, and after unfolding, finally degrades the substrate (MAPKKK18). Dephosphorylation of its specific amino acid residues (S/T) by the phosphatase ABI1 is another known signal for MAPKKK18 degradation.

MAPK signaling pathways are involved in signal transduction during plant growth, development, and adaptation to various stresses. However, the strength and duration of MAPK activation must be tightly controlled to achieve the correct biological responses. Therefore, the activation and inactivation of the individual components of the MAPK pathway are also of interest. Both MAPKKK18 protein levels and activity are carefully maintained by internal phytohormone signals and external environmental cues. Previous studies have implicated the core ABA signaling pathway in the regulation of MAPKKK18 abundance and activity. MAPKKK18 phosphorylation, activation and de novo protein synthesis are regulated by PYR/PYL/RCAR-SnRK2 module after ABA treatment (Danquah et al. 2015, Mitula et al. 2015, Tajdel et al. 2016). For example, the ABA core pathway activates the MAPKKK18-MKK3-MPK1/2/7/17 module through transcriptional regulation of MAPKKK18, resulting in MAPKKK18 activation (Danquah et al. 2015, Matsuoka et al. 2015, Mitula et al. 2015). The expression of MAPKKK18 is also regulated by a key transcription factor in ABA signaling, ABI4, in the process of ABA-induced stomatal closure. As a result of ABA-induced persulfidation of ABI4, ABI4 can bind to the promoter of MAPKKK18 (Zhou et al. 2021). In addition, very recent results by Zhao et al. (2023) showed that three other TFs, ABA-responsive element binding factors 2, 3 and 4 (ABF2/3/4), act as upstream transcription factors of MAPKKK18 expression in the process of leaf senescence. This regulation of MAPKKK18 transcription by components of the ABA signaling pathway appears to be critical for plants under stress, forming feedback loops to achieve sustainable adaptation (Zhao et al. 2023).

In addition to positive regulation, negative regulation is also important for plants to achieve appropriate biological responses and prevent sustained activation of the MAPK pathway. However, only a few examples of negative regulation of MAPK cascades have been characterized and described. The most common mechanisms include dephosphorylation by phosphatases and proteasomal degradation. Indeed, on the one hand, our previous studies have shown that in the absence of ABA, MAPKKK18 is directly dephosphorylated by ABI1, which not only inhibits MAPKKK18 kinase activity but also plays a predominant role as a signal for proteasomal degradation of MAPKKK18 (Ludwików 2015, Mitula et al. 2015). On the other hand, ABA binding to PYR/PYL/RCAR receptors blocks MAPKKK18 turnover via the proteasome pathway, allowing MAPKKK18 to activate downstream substrates (Mitula et al. 2015). In this work, we revealed new key players in the mechanism of MAPKKK18 proteasomal degradation. We have successfully identified three E3 ligases: KEG, UPL1 and UPL4, which negatively regulate MAPKKK18 accumulation and target MAPKKK18 for proteasomal degradation (**Supplementary Tab. 1**, **Figs. 1**, **2**). Interestingly, recent studies show that two other RING-type ligases, RGLG1 and RGLG2, are also able to ubiquitinate MAPKKK18 and promote its degradation (Yu et al. 2021). UPS-mediated degradation of many other proteins is known to involve more than one E3 ubiquitin ligase (Deshaies and Joazeiro 2009). This includes ABA effectors, e.g. the regulation of the phosphatase PP2C ABI1 ubiquitination involves at least three types of E3 ligases—PUB12/13 (Kong et al. 2015), CRL3 (Julian et al. 2019), AIRP3 (Pan et al. 2020). It is therefore possible that specific ligases that modify MAPKKK18 are closely linked to particular stages of plant development or different processes. Since RGLG1/2 have been shown to mediate drought stress tolerance (Yu et al. 2021), it may be worthwhile to conduct further studies to identify specific processes in which MAPKKK18 kinase abundance is regulated by individual ligases, including KEG, UPL1 and UPL4. Of these, the E3 ligase KEG is known to be a negative regulator of ABA signaling; *keg* plants display an ABA hypersensitive and retarded growth phenotype (Stone et al. 2006, Liu and Stone, 2010). KEG affects the abundance of the ABA-responsive transcription factor ABI5 (Stone et al. 2006), two other ABI5-related transcription factors, the ABA-binding factors ABF1 and ABF3 (and CIPK26 kinases (Lyzenga et al. 2013). Furthermore, recent studies have identified MAP kinases, MAPKK4 and MAPKK5 as KEG substrates. Results from Gao et al. (2020) indicate that both kinases are ubiquitinated by KEG and associate only with the full version of the KEG protein, while no interactions with its fragments were reported. These results are inconsistent with our results showing that MAPKKK18 is able to associate (not only with full-length KEG), but also with truncated KEG-RKA and KEG-H (**Fig. 1B, C**). However, it should be emphasized that the HERC domain in the KEG protein is required for interaction with the MAPKKK kinase EDR1 (Gu and Innes, 2011), and the ARM domain normally mediates E3 ligase interaction with substrate proteins (Kong et al. 2015, Saini et al. 2023). Since keg loss-of-function mutants are lethal, to test whether KEG targets MAPKKK18 for proteasomal degradation, we assayed the degradation of MAPKKK18 in cell-free extracts prepared from mutants generated by silencing KEG using an engineered microRNA approach (Pauwels et al. 2015). The results obtained showed that the degradation rate of MAPKKK18 was delayed compared to the degradation rate in wild plant extracts (**Fig. 1D, E**). We therefore suggest that RING E3 ligase KEG controls MAPKKK18 turnover.

Together with KEG ligase we also investigated the role of two HECT E3 ligases, UPL1 and UPL4, in regulating MAPKKK18 stability. In *A. thaliana*, seven different members of HECT E3 ligases have been identified and grouped into four subfamilies (UPL1/2, UPL3/4, UPL5 and UPL6/7) (Downes et al. 2003, Cho et al. 2017). In *Arabidopsis* single knockout plants, UPL1, UPL3, UPL4 and finally UPL5, a significant decrease in the cellular level of ubiquitin conjugates has been found (Furniss et al. 2018; Wang et al. 2023).Therefore, it is speculated that E3 HECT ligases are ligases that are much less specific than, for example, RING ligases and therefore have a much larger pool of substrates (Wang and Spoel 2022). Importantly, the role of UPL1 and UPL4 in plants is not well characterized or are their substrates. To date, only one protein ubiquitinated by UPL4 has been identified. It has been shown that UPL4 acts with its closest paralog, UPL3, in the polyubiquitination of the transcriptional co-activator of plant immunity, NPR1, and is therefore critical for proteasomal clearance from promoters of NPR1 target genes (Wang et al. 2023). UPL3/4 may also be involved in controlling developmental processes such as hypocotyl elongation, apical hook formation and root growth by assisting the proteasome in degrading EIN3 (Wang et al., 2022). It is also known that UPL1 and UPL4, together with UPL3 and UPL5, are plant immune regulators that regulate salicylic acid (SA)-responsive genes (Furniss et al. 2018). In the present work, we successfully identified the direct interaction between MAPKKK18 and UPL4 (200 kDa) (**Fig. 2D**). Unfortunately, due to the large size of the UPL1 protein, approximately 405 kDa, it was not possible to perform such an analysis with full-length UPL1. However, interaction analyses were performed with fragments of the UPL1 protein, but no interaction was reported (**Fig. 2B**), suggesting that the nature of the UPL1–substrate interaction is complex and most likely determined by the mutual spatial orientation and configuration of the interacting components. All E3 HECT ligases consist of a conserved C-terminal HECT domain, which is involved in the ubiquitination of UPL substrates, and distinct N-terminal domains (Furniss et al. 2018; Wang and Spoel; 20–22). Like UPL4, UPL1 not only contains a domain with an ARM repeat, but it is also composed of a UBA domain and contains three domains of unknown function (**Fig. 2A**; Lan et al. 2018). Thus, it is very likely that the full version of UPL1, or at least fragments composed of several domains, are required for the interaction, but the size of the protein is still a limitation for these analyses. However, the results of the cell-free degradation experiment clearly show that MAPKKK18 degradation is significantly delayed in extracts lacking these ligases, so we can conclude that UPL1 and UPL4 affect the stability of the MAPKKK18 protein (**Fig. 2E, F**).

The ubiquitination process is highly conserved and is based on the covalent attachment of the ubiquitin molecule to specific lysine residues in the target protein (Pickart, 2001; Hua and Murray, 2023). To explore the molecular mechanism of MAPKKK18 proteasomal degradation, based on prediction with available online tools and relevant molecular modeling of ubiquitin acceptor sites, we examined the degradation ratio of several mutant versions of GST–MAPKKK18. Here we identified two lysins 154 and lysine 237, whose ubiquitination is a signal for degradation (**Figs. 4**, **5**). These results are partially consistent with those of Yu et al. (2020), who also implicated lysine 154 in the process of proteasomal degradation of MAPKKK18.

Interestingly, this study investigates the role of the ubiquitin-proteasome pathway in regulating the close paralog of MAPKKK18—MAPKKK17. We have shown that MAPKKK17 is also degraded by the 26S proteasome and identified key E3 ligases involved in this process (**Fig. 6**). MAPKKK17 shares very similar expression characteristics with MAPKKK18 in different tissues and in response to different stimuli (Li et al. 2017). It is also known that the kinase activity of MAPKKK17 is significantly increased after ABA treatment (Danquah et al. 2015). Importantly, MAPKKKK18 and MAPKKK17 recruit the same downstream module MKK3-MPK1/2/7/14 to mediate the ABA-mediated response and are regulated by the same E3 ligases, supporting redundancy and the coordinated regulation of both kinases in the ABA response.

In conclusion, we have identified three E3 ligases that can ubiquitinate MAPKKK17/18, the RING ligase KEG and two UPL ligases UPL1 and UPL4. We also identified two lysine residues in the MAPKKK18 sequence that modulates its turnover, lysine 154 and lysine 237. It remains to be verified which ligase catalyzes the attachment of ubiquitin to lysine 154 and 237. It is also necessary to determine the specificity of this ligase in modifying certain MAPKKK17/18 lysine residues and to identify the process by which this specific modification of MAPKKK17/18 occurs.

## Materials and Methods

### Plant material and growth conditions

Sterilized *A. thaliana* seeds were sown on peat discs or on solid half-strength MS medium plates supplemented with 1% sucrose. After 3 days of seed stratification at 4°C, plants were grown in growth chambers under the following growth conditions: temperature 21°C, humidity 70%, irradiance 150–200 µE/m^2^s (16 h light and 8 h dark). The WT *A. thaliana* natural accession Col-0 (Columbia; WT Col-0) was used. Mutant seeds of *upl1* (SALK_063972C) and *upl4* (SALK_105288C) were obtained from the European *Arabidopsis* Stock Center. Seed od *mapkkk17* were obtained from dr Jean Colcombet (Salk_137069). The KEG ami3 and ami14 lines we obtained from dr Laurens Pauwels and has been described previously (Pauwels et al. 2015).

For the germination assay, *Arabidopsis* seeds were surface-sterilized with 3%NaClO solution for 10 min, washed twice in 96% ethanol and dried. The seeds were then sown on 0.8% agar plates containing ½ MS medium supplemented with or without 0.5 μM (+) ABA (Duchefa Biochemie, Haarlem, Netherlands). After 3 days of stratification at 4°C in the dark, the plates were transferred to the growth chamber. Germination, defined as fully expanded cotyledons, was scored after 7 days.

### Mass spectrometric analysis

GST–MAPKKK18 was incubated with total plant extract isolated from 7-d-old *A. thaliana* WT-Col 0 seedlings using isolation buffer containing 1× PBS pH 7.4, 0.1 metre NaCl, 0.1% NP-40, 1 mM DTT, 1 mM PMSF and protease inhibitor. After several washes according to the recommendations of the resin manufacturer (GE Healthcare), the samples were sent on dry ice for mass spectrometric analysis at the Mass Spectrometry Laboratory of the Institute of Biochemistry and Biophysics of the Polish Academy of Sciences in Warsaw (IBB, Warsaw, Poland). A tandem spectrometer, Thermo Orbitrap Velos (ThermoScientific, Dreieich, Germany, was used for the analyses. The spectrometer software then selected specific peaks from the mass spectra, eliminating the extreme peaks. The commercial Mascot identification system Mascot Distiller software (version 2.4.2.0, MatrixScience) was used to check The *A. thaliana* TAIR10 database (35,386 sequences; 14,482,855 residues).

### Vector construction

The complete coding sequences of MAPKKK17, MAPKKK18, UPL4 and deletion fragments of UPL1 and KEG E3 ligases were amplified by PCR using the specific primer pairs (**Supplementary Table 3**) and ligated into the pENTR-SD/D-topo vector (Invitrogen, Carlsbad, CA, USA). The KEG cDNA clones were obtained from dr Judy Callis. Mutated versions of MAPKKK18 in the pENTR vector (MAPKKK18 mutants: K7R; K7R/K32R; K7R/K32R/K146R; K7R/K32R/K146R/K154R; K237 R; K154R) were generated using the QuikChange II XL Site-Directed Mutagenesis Kit (Agilent, Santa Clara, CA, USA) by PCR with primers designed for a specific mutation (**Supplementary Table 3**). To generate expression vectors (encoding N-tagged or C-tagged protein fusions), MAPKKK17/18 or E3 ligase sequences in the pENTR vector were recombined with destination vectors, including Gateway® pDEST™15 (Invitrogen), Gateway® pDEST™17 (Invitrogen), Champion™ pET300/NT-DEST (Invitrogen), pSITE4NB, pSITE2CA (Chakrabarty et al. 2007) using Gateway®LRTo Clonase® II enzyme mix (Invitrogen), modified pSAT3-cCFP-DEST (pS6C1C; Mitula et al. 2015) and the pSAT5-DEST-nVenus (pS5N4N; Mitula et al. 2015). Both sequencing and restriction digestion with the appropriate enzyme(s) were used for final verification.

### In vitro protein expression and purification

Individual expression vectors producing GST- or HIS-tagged recombinant proteins (including GST–MAPKKK18 and its mutated versions, GST–MAPKKK17, HIS-MAPKKK18, GST-KEG-RKA, GST-KEG-RK, GST-KEG-H) were transformed into chemically competent *Escherichia coli* B One Shot™ BL21(DE3)pLysS (Invitrogen). For protein overexpression, single colonies were inoculated into 20 ml LB media with ampicillin and chloramphenicol for positive selection and grown overnight at 37°C. Starter cultures were used to inoculate 100 ml of fresh LB media supplemented with the appropriate antibiotics and grown at 37°C with continuous shaking (180 rpm) to achieve OD600 0.6. Expression of the recombinant proteins was induced by 0.5 mM isopropyl-β-d-1-thiogalactopyranoside. After ∼4 h of induction, bacterial cells were harvested by centrifugation, resuspended in PBS with the addition of phenylmethanesulfonyl fluoride and then lysed in a probe sonicator (Sonics Vibra Cell, Sonics and Material Inc, USA). Total lysates were centrifuged at 4°C and the supernatant was incubated with Ni-NTA resin (Invitrogen) or glutathione-Sepharose 4B resin (GE Healthcare, Chicago, Illinois, USA) for ∼1 h and washed according to the manufacturer’s protocol.

### Pull-down assay

Pull-down experiments were conducted using specific bait proteins fused to GST-tag using procedures described in Hagemeier et al. (1993), Mitula et al. (2015) and Marczak et al. (2020). GST-KEG fragments were bound to Glutathione-Sepharose 4B resin, and then to prevent nonspecific binding to the resin, incubated 10 min at RT with bovine serum albumin (BSA, 1 mg/ml). Then, prey protein—HIS-MAPKKK18 and EBC buffer (containing 100 mM NaCl; 0.5% 100 mM NaF; 200 μM sodium orthovanadate; Nonidet P-40; 50 mM Tris–HCl pH 8.0) were added to the final volume of 100 μl. After 1 h incubation at RT, resin was washed using NENT buffer, with increasing NaCl concentration (100 mM/200 mM/300 mM NaCl; 1 mM EDTA; 0.5% Nonidet P-40; 20 mM Tris–HCl pH 8.0). Then 5×SDS–PAGE sample buffer was added, and samples were boiled 10 min in 98°C. After electrophoretic separation of proteins in the presence of SDS, proteins were transferred to a polyvinylidene difluoride (PVDF) membrane and then analyzed by immunoblotting.

### Transient expression in protoplasts

Protoplast used for BiFC and FRET analysis were isolated from 3-week-old *A. thaliana* WT Col-0 leaves and transformed as described in Ludwików et al. (2014), Mitula et al. (2015). Protoplasts were observed using a Nikon A1R confocal microscope with Plan Apo 20/40 objectives. To excite eGFP fluorescence, a 488 nm laser was used, and a 500–550 nm emission filter was used for detection. A 561 nm laser was used to excite mRFP fluorescence and a 570–620 nm emission filter was used to capture the fluorescence. Nikon NIS-Elements software was used for data documentation. In the multicolor bimolecular fluorescence complementation assay (mcBiFC), a mixture of plasmids encoding pairs of analyzed proteins in fusion with cGFP and n-Venus was used for transformation (maximum 10 μg of plasmid mixture per 100 μl of protoplast). Transformed protoplasts were incubated overnight in growth chambers.

### Cell-free degradation assay

The cell-free degradation assay was performed as reported in Ludwików et al. (2014), Mitula et al. (2015) and Janicki et al. (2020). Briefly, 300 µg of recombinant GST–MAPKKK17/18 or its mutated forms were incubated with 500 µg of plant extract isolated using buffer containing 10 mM NaCl; 5 mM DTT; 4 mM PMSF; 20 mM Tris–HCl pH 7.5, in the presence of ATP at a final concentration of 20 μM. The reaction was stopped by adding SDS-PAGE loading buffer and freezing. GST–MAPKKK/18 was detected by immunoblotting using anti-MAPKKK18 (1:5000; Agrisera, Vännäs, Sweden). GST–MAPKKK17 was detected by immunoblotting using anti-GST(1:5000, cat nr. GSTT01, MoBiTec, Goettingen, Germany). Ponceau S staining or immunoblotting with anti-actin antibody (1:4000; Cat nr. Merck, Darmstadt, Germany) was used as a loading control.

### Immunoblotting

Prior to loading protein samples onto the 10% SDS–PAGE gel, 5×x SDS–PAGE loading buffer was added and samples were boiled at 98°C for 10 min. After appropriate electrophoretic separation, proteins were transferred to an Immobilon®-P PVDF membrane (Merck Millipore). The activated membranes were blocked with 5% blocking agent (skim milk) diluted in PBS-T buffer (pH 7.4). The blocked membranes were washed three times with PBST buffer with gentle shaking for 5 min. The membranes were then incubated with primary antibodies, including anti-MAPKKK18 (1:5000, cat nr. AS13 2673, Agrisera), anti-GST (1:5000, cat nr. GSTT01, MoBiTec, Goettingen, Germany), anti-HIS (1:1000 cat nr. HIST01, MoBiTec), anti-actin (1:4000, cat nr. A0480, Merck). After three washes as before, the membranes were incubated for 1 h with secondary antibody anti-rabbit (1:35 000; Agrisera) diluted in 5% blocking solution (skim milk) in PBS-T. ECL (ThermoFisher Scientific) was used for detection. ChemiDoc Imaging System (Biorad) was used for imaging and documentation.

### Modeling and preparation of protein structures

Models of Ub and MAPKKK18 protein structures were developed using the AlfaFold2 (Jumper et al. 2021). The best structure model was used for further analysis. The obtained structures were prepared and minimized in the OPLS4 force field (Lu et al. 2021) using the Preparation Wizard protocol implemented in Schrodinger Suite 2022 (Sastry et al. 2013).

### Molecular dynamics

The Desmond (Desmond 2022) software package was used for molecular dynamics simulation. A Nose–Hoover thermostat and Martyna–Tobias–Klein barostat were used in each system’s NPT ensemble class simulation at 300 K and a constant pressure of 1 bar. The integration time step was 2 fs. The total simulation time was 100 ns. The hydrogen-heavy atomic bonds were kept rigid using the SHAKE algorithm. A smooth-particle Ewald mesh was used for long-range Coulomb interactions, while a cutoff of 9 was set for short-range Coulomb interactions. Structures were prepared and minimized prior to simulation. Using the TIP3P water model, each system was solvated in an orthorhombic box after charges were balanced and 0.15 metre NaCl was added. The OPLS4 force field settings were used in each simulation. Desmond tools were used for post-processing analysis including clustering, rmsd and rmsf calculations.

### Protein–protein docking studies and scoring

The Cluspro web server was used for initial docking studies (Kozakov et al. 2017). The blind docking protocol was followed. No constraints were applied during the protein–protein docking. The resulting complex models were ranked according to balanced schema. In the next step, the Haddock (Van Zundert et al. 2016, Honorato et al. 2021) was used and the calculations were performed with adding constraints and default settings. In this step, the single lysine of the target protein and G76 of Ub were selected as active residues during the macromolecular docking studies. The obtained results were ranked based on haddock scoring function, distance between each lysine of MAPKKK18 and G76 of Ub and binding energy calculated by Molecular Mechanics-Generalized Born Surface Area (MM-GBSA) method using Prime module (Jacobson et al. 2004). The value of the binding energy was calculated as the difference between the energy of the MAPKKK18–Ub complex and the energy of the unbound MAPKKK18 and Ub. The entropy term was neglected. The Variable-Dielectric Generalized Born solvation model (Li et al. 2011) was used in the OPLS4 force field (Lu et al. 2021) with a minimized sampling method. The evaluated structures were analyzed and visualized using PyMOL (DeLano 2002).

## Supplementary Material

pcad165_Supp

## Data Availability

The article and the online supplementary data contain all the data on which this article is based. Materials used in this study are available from the corresponding author upon request.
