# Supplementary material for: Arabidopsis HECT and RING-type E3 Ligases Promote MAPKKK18 Degradation to Regulate Abscisic Acid Signaling
Source: Plant Cell Physiol. 2023 Dec 28;65(3):390–404. doi: 10.1093/pcp/pcad165 (PMC11020294; doi:10.1093/pcp/pcad165)
Supplement: pcad165_Supp [file pcad165_supp.zip › supp/pcp-2023-e-00201-File010.pdf]

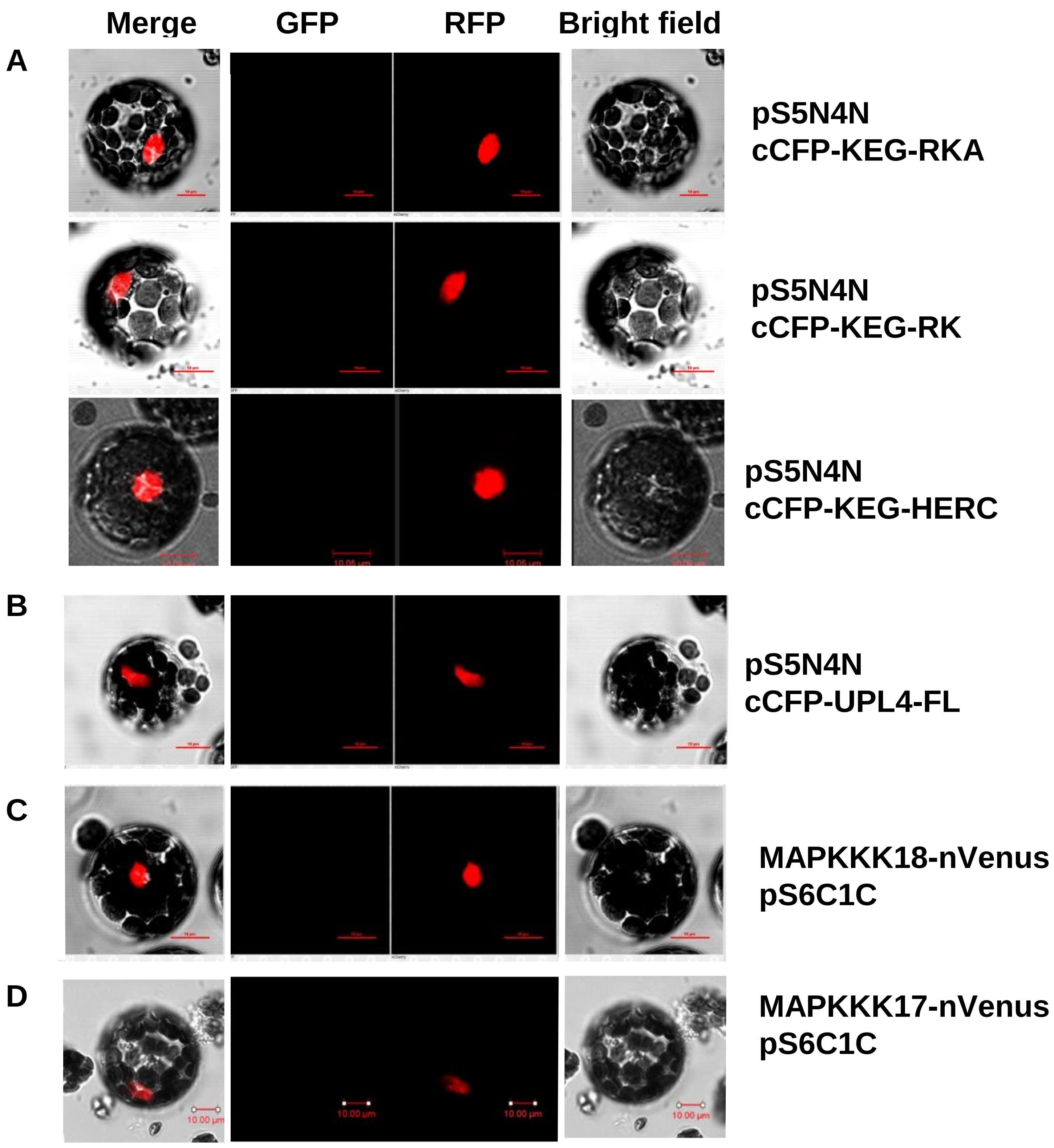


**Supplementary Figure 1. Negative controls for a multicolour bimolecular fluorescence complementation (mcBiFC) experiment.**

**A.** Negative controls were performed using Arabidopsis protoplasts cotransformed with individual K EG fragments fused to cCFP (cCFP-KEG-RKA, cCFP-KEG-RK and cCFP-KEG-H) and the empty vector encoding nVenus (pS5N4N). **B.** Protoplast transformed with construct pairs: cCFP-FL-UPL4 with empty vector encoding nVenus. **C.** Arabidopsis protoplasts cotransformed with the empty vector encoding cCFP (pS6C1C) and MAPKKK18-nVenus. **D.** Cotransformation of protooplast with the empty vector cCFP (pS6C1C) and MAPKKK17-nVenus. No BiFC signal was observed in the GFP channels. Scale bar, 10 µm.
