# Supplementary material for: Arabidopsis HECT and RING-type E3 Ligases Promote MAPKKK18 Degradation to Regulate Abscisic Acid Signaling
Source: Plant Cell Physiol. 2023 Dec 28;65(3):390–404. doi: 10.1093/pcp/pcad165 (PMC11020294; doi:10.1093/pcp/pcad165)
Supplement: pcad165_Supp [file pcad165_supp.zip › supp/pcp-2023-e-00201-File011.pdf]

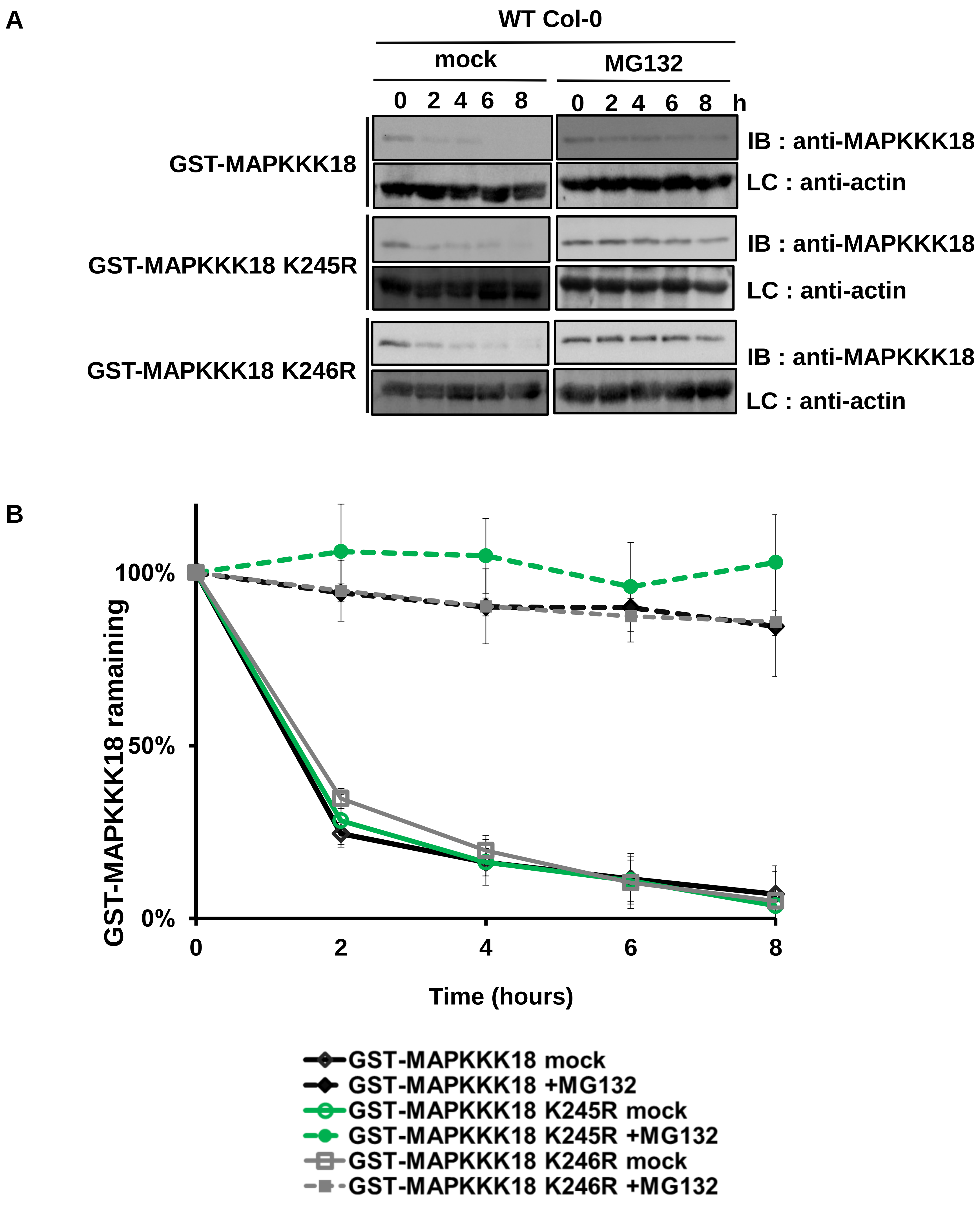

Supplementary Figure 2. Lysine 245 and 246 not involved in MAPKKK18 proteasomal degradation.

A.Cell-free degradation assay of site-specific GST-MAPKKK18 mutants K245R and K246R in WT plants. GST-MAPKKK18K245R, GST-MAPKKK18K246R and GST-MAPKKK18 proteins were incubated with WT Col-0 total plant extract in the presence of ATP, with and without MG132. The levels of each recombinant protein were checked by Western blotting using specific anti-MAPKKK18 antibodies at the indicated time points. B. The amount of GST-MAPKKK18 and its mutant versions was determined by ImageJ using the signal intensity at the indicated time and plotted. Error bars indicate the standard deviation.
