# Supplementary material for: Arabidopsis HECT and RING-type E3 Ligases Promote MAPKKK18 Degradation to Regulate Abscisic Acid Signaling
Source: Plant Cell Physiol. 2023 Dec 28;65(3):390–404. doi: 10.1093/pcp/pcad165 (PMC11020294; doi:10.1093/pcp/pcad165)
Supplement: pcad165_Supp [file pcad165_supp.zip › supp/pcp-2023-e-00201-File012.pdf]

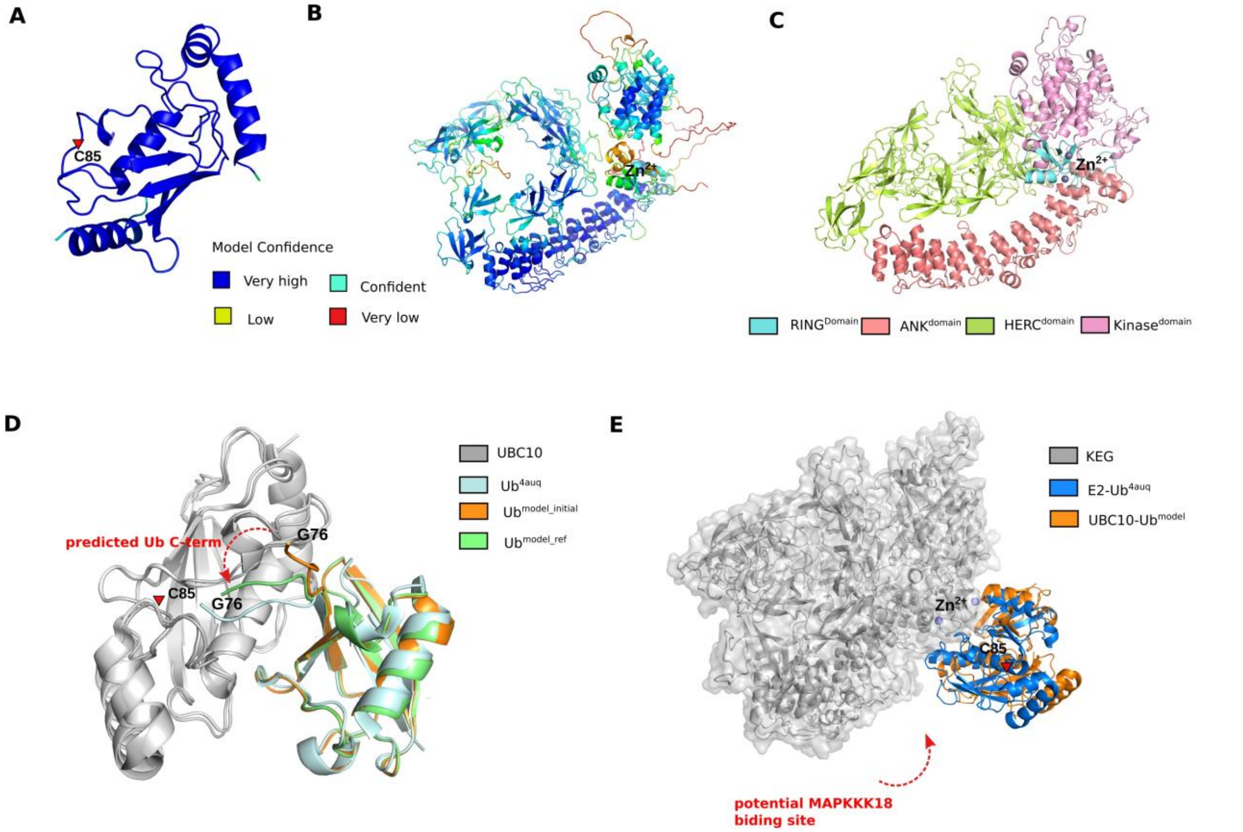

Supplementary Figure 3. Molecular modelling of the UBC10-Ub-KEG (E2-Ub-E3) complex.

AF2 prediction of UBC10 structure (E2 ligase) with marked C85 residue (A) and KEG structure (E3 ligase) with marked ZN2+ binding motif (RING domain) (B). The quality of the obtained structures was coloured according to the model confidence (pDLLT). A higher value of pLDDT indicates a higher local quality of the model, pLDDT value ranges from 0 to 100 (A, B). KEG ligase structure with marked domains: RING, kinase domain, ankyrin repeats (ANK) and HERC domain. This structure model was obtained after 100ns MD simulation, post-processing stage including clustering and minimisation. The structure obtained after post-processing was used for further analysis (C). The best obtained model of the Ubc10 (E2)-Ub complex, aligned with the E2-Ub structure from the experimental structure (pdb code: 4auq) (Dou et all, 2012). Prediction of Ub N-term conformation from MD data and refinement compared with experimental orientation of Ub N-term in E2-Ub complex from pdb structure (4auq). Residues C85 and G76 are labelled. The interaction interface from 4auq was used for docking. (D) Top binding pose of UBC10-Ub obtained during docking of UBC10-Ub to KEG ligase, compared to the reference orientation of E2-Ub from pdb structure 4auq. The orientation of E2-Ub was based on the structural alignment of the 4auq structure and the KEG RING domain (10-56 aa). To construct the KEG-UBC10-Ub complex, the interaction interface between the RING domain and the E2 ubiquitin conjugate structure (4auq) was used (E). The predicted KEG-UBC10-Ub complex was used in further analysis to verify the kinase MAPKKK18 binding site. Models of UBC10-UB and UBC10-Ub-KEG were predicted using Haddock macromolecular docking software (D, E). All results were ranked according to Haddock score and compared with structural data (pdb code 4auq) by visual analysis.

Citation

Birc7-E2 Ubiquitin Conjugate Structure Reveals the Mechanism of Ubiquitin Transfer by a Ring Dimer. Dou, H., Buetow, L., Sibbet, G.J., Cameron, K., Huang, D.T.(2012) Nat Struct Mol Biol 19: 876.
