# Supplementary material for: Arabidopsis HECT and RING-type E3 Ligases Promote MAPKKK18 Degradation to Regulate Abscisic Acid Signaling
Source: Plant Cell Physiol. 2023 Dec 28;65(3):390–404. doi: 10.1093/pcp/pcad165 (PMC11020294; doi:10.1093/pcp/pcad165)
Supplement: pcad165_Supp [file pcad165_supp.zip › supp/pcp-2023-e-00201-File013.pdf]

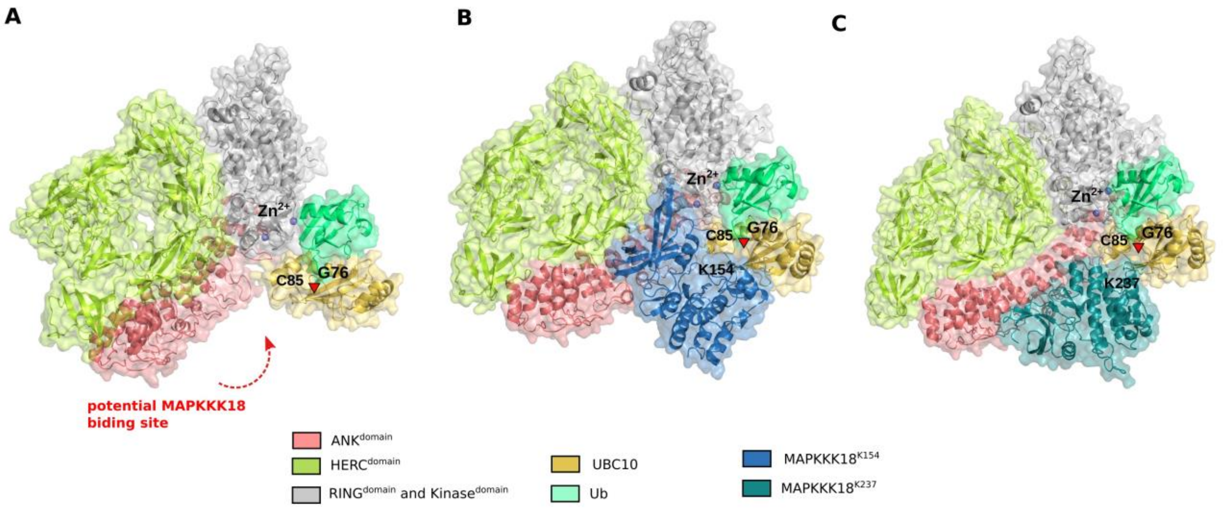

Supplementary Figure 4. UBC10-Ub-KEG-MAPKKK18 complex prediction using multistep macromolecular modelling.

KEG-UBC10-Ub potential complex, obtained from macromolecular docking, with marked C85, G76, ZNn2+ (RING domain), ANK repeats and HERC domain. Possible MAPKKK18 binding site to allow Ub molecule transfer (A) Obtained best models of UBC10-Ub-KEG-MAPKKK18^{K154} (B) and UBC10-Ub-KEG-MAPKKK18^{K237} (C) with marked C85, G76 and two identified lysine residues (K154, K237) as amino acids involved in Ub covalent attachment to MAPKKK18. Obtained models revealed that MAPKKK18 interacts with two domains of KEG E3 ligase: HERC domain and ANK repeats (B, C). To construct UBC10-Ub-KEG-MAPKKK18 complex KEG-UBC10-Ub model (A) and AF2 predicted MAPKKK18 structure (C-term was neglected) was used. In this prediction two approaches were used. First, we use K154 from MAPKKK18 and C85 from KEG-UBC10-UB complex as active residues during docking protocol. In second approach we follow similar procedure but K237 was marked as active residu. Structure complexes of UBC10-Ub-KEG-MAPKKK18 were predicted using Haddock. All obtained models were ranked according to Haddock score and followed the visual inspection to check possibility of forming covalent bond between selected lysine residue (K154, K237) and G76 (distance between G76 and K residue). After this analysis models that were unable to form a covalent bond were rejected. Last step was selection of models based on experimental data showed in this study that MAPKKK18 can interact with ANK repeats and HERC domain.

Citation

Birc7-E2 Ubiquitin Conjugate Structure Reveals the Mechanism of Ubiquitin Transfer by a Ring Dimer.
Dou, H., Buetow, L., Sibbet, G.J., Cameron, K., Huang, D.T.(2012) Nat Struct Mol Biol 19: 876
